# Textual health warning labels on snus (Swedish moist snuff): do they affect risk perception?

**DOI:** 10.1186/s12889-018-5461-2

**Published:** 2018-04-27

**Authors:** Connie Villemo Nilsen, Oddgeir Friborg, Karl Halvor Teigen, Frode Svartdal

**Affiliations:** 1 Department of Psychology, Faculty of Health Sciences, UiT The Arctic University of Norway, 9037 Tromsø, Norway; 2 Department of Psychology, Faculty of Social Sciences, University of Oslo, P.b. 1094, Blindern, 0317 Oslo, Norway

**Keywords:** Smokeless tobacco, Swedish moist snuff, Snus, Warning labels, Risk perception, Tobacco control

## Abstract

**Background:**

To strengthen the risk message on snus warning labels, the European Union in 2016 removed “*can”* from the warning “*This tobacco product (can) damages your health and is addictive.”* We tested how these and other textual warnings affect risk perception.

**Methods:**

Snus-using and non-using Norwegians aged 16–72 participated in two online survey experiments. Participants in Study 1 (*N* = 196) were randomized to read one of four warning labels. Outcome variables included ratings of likelihood of health damage from snus and perceived severity of such damages. Study 2 (*N* = 423) used similar outcome measures but added a baseline measure allowing for a pre-post comparison, as well as a control group receiving no warning label. Data were analysed using ANOVA and non-parametric tests.

**Results:**

Study 1 indicated that removing “*can*” from the EU warning increased long-term risk perception, but adding “*causes cancer”* had no effect on risk perception. In Study 2, risk perception increased from pre to post, regardless of label manipulation. “*Causes cancer”* and “*damages your health”* were indicated as most alarming when participants compared and ranked all warnings.

**Conclusions:**

Adding “*causes cancer*” or removing “*can”* from “*damages your health”* did not strengthen short-time (1 year) risk perception, but the latter increased long-term (10 years) risk perception in Study 1. In the pre-post design in Study 2, risk perception increased regardless of warning label.

## Background

The use of snus (a moist oral smokeless tobacco product) has been increasing in Norway, especially among young people aged 16–24. The number of daily and occasional young users increased from 9% of males and 2% of females in 2003 to 33% and 23% in 2013 [1], an increase considered as “almost an epidemic” by the Norwegian Institute of Public Health [1]. This report concluded that snus is associated with several health risks, such as lesions of the oral cavity, adverse pregnancy outcomes, and some forms of cancer. Although snus is considered as less harmful compared to smoking [2], the risks associated with snus use should be communicated to users and potential new users. One way of informing the public is by the use of product warning labels.

Whereas the effect of warning labels for smoked tobacco has been thoroughly researched, comparable studies on smokeless tobacco (SLT) are scarce [3]. The literature for snus specifically is even more limited. Hence, the present summary includes SLT labels in general, and implications for snus warnings should be interpreted carefully, as health risks from snus differ from other SLT products. Mere *textual warnings* seem to be noticed and remembered, but their effect on intentions to use SLT is small [4]. In this study, around 40% of the adolescents exposed to textual warnings recalled seeing a warning label, and of these one in three remembered the content of the warning. Males remembered the warnings somewhat better than females, which is reasonable as males tried or purchased such products more frequently than women did. However, remembering warnings did not reduce future intentions of using SLT. MacKinnon and Fenaughty [5] found that heavy SLT users remembered written warnings better than non-users, possibly due to repeated exposure. In a 2016 study [6], about four in five users remembered exposure to textual warning labels, and recall was closely associated with self-reported thoughts about health risks and perceived harmfulness of SLT. Still, less than one in five said warning labels had stopped them from using SLT on some occasion.

In comparison, *graphical warnings* seem to have a greater effect in capturing attention and motivating smokers to quit [7]. One study [8] found that pictorial versions evoked more concerns about health risks compared to mere textual ones, and pictorial versions were judged as least attractive to SLT users whereas textual warnings were seen as more appealing for peers (i.e., the kind of package a peer would want to be seen using). In contrast, another study found no increase in risk perception in a sample of non-users who were shown graphic cancer warnings on snus products [9]. It is worth noting that the baseline risk perception in this sample was high, possibly preventing a further increase (ceiling effect).

Whereas graphical warning labels are mandatory for smoked tobacco in the European Union (EU), the requirement for snus products is limited to textual warnings. In 2003, EU removed the warning “*causes cancer”* [10] from snus products, and replaced it with the more general warning: “*This tobacco product can damage your health and is addictive”* [11]. It can be assumed that the previous “*causes cancer*” warning was more alarming than the more general claim “*damages your health,*” but to our knowledge there is no evidence supporting this expectation. In May 2016 the warning message was strengthened by removing the modal verb “*can*” [12], following an EU-directive adopted in 2014 [13].

Whereas the EU-expectation of a strengthened risk message by removing *can* gained support in focus group interviews among SLT users [14], the effect of removing *can* may be more complex. Specifically, Teigen and Filkuková [15] found that statements including *can* were associated with an outcome being possible, but uncertain, whereas *will-*statements were perceived as referring to more probable or certain outcomes. Moreover, *can* evoked expectations of high magnitude effects, whereas *will* denoted low to medium effects [15]. According to this line of reasoning, removing *can* might reduce severity perceptions but increase expected likelihood of health damage from snus.

In light of these findings, we examined risk perception from snus warning labels in Norway. Although the verbs *can* and *will* are frequently used on warning labels, we could not identify any studies comparing possible effects of this difference. As the EU changed these particular verbs on snus warnings in 2016, it is of interest to examine whether they differentially affect risk perception. Specifically, we examined the following hypotheses:

- H1: In line with the EU-directive 2014/40/EU, removing *can* from *damages your health* will strengthen the risk message.
- H2: Removing *can* from *damages your health* should decrease severity expectations, but increase likelihood perception, in line with Teigen and Filkuková [15].
- H3: As the *can*/*will* labels target general health only, a warning explicitly stating that snus *will* severely damage health and *cause cancer* (i.e., the EU warning before 2003) should generate stronger risk expectation than either of the other labels.

Risk perception from textual warning labels on snus products was tested in two separate studies. In Study 1, participants read one of four warning labels and then responded to risk perception measures. Study 2 added a baseline for outcome measures, a control group seeing a snus product with no warning, and an expert panel responding to the same measures. As most of the warning labels also include an assertion about ease of addiction (see Table 1), we added a rating related to ease of addiction. Both studies were conducted before the modal verb *can* was removed from the snus warning message “*This tobacco product (can) damage your health and is addictive*” [13].

**Table 1:** Textual content of warning labels

EU implementation	Abbr.	Warning label
1. ➔ 2003	Cancer	“This tobacco product severely damages your health. Causes cancer.”
2. Not applied	Can-can	“This tobacco product can damage your health and be addictive.”
3. 2003–2016	Can-is	“This tobacco product can damage your health and is addictive.”
4. 2016 ➔	Will-is	“This tobacco product damages your health and is addictive.”

### Study 1

#### Participants

The total sample was 196 participants (151 female, 6 did not indicate gender), age 16–64 years (*M* = 34.14, *SD* = 10.70). Participants were recruited through snowballing in social media (www.facebook.com), January 2016. All completed an online questionnaire (www.qualtrics.com) following electronic informed consent. Participants not understanding Norwegian language or being < 16 years of age were excluded. There were no incentives for participation.

#### Materials and procedure

**Fig. 1:**
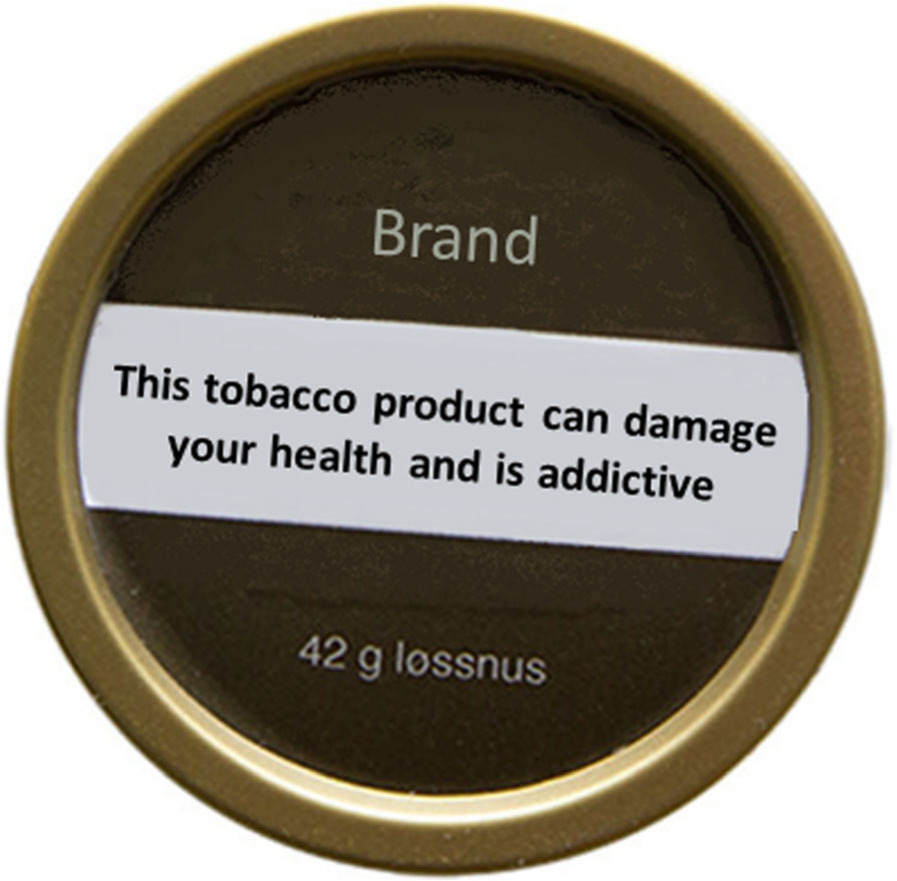
Example of snus product with a textual warning label_1._ ‘Løssnus’ means loose snus (non-pouched)

Participants were randomly assigned to read one of four warning labels on a brand-neutral snus product, thus making the text scenario realistic. Figure 1 presents an example,^1^ and Table 1 summarizes the textual warnings. The questionnaire then asked about expected severity of health damages following use of snus, likelihood for such health damages after one and ten years, and perceived ease of addiction. Demographic data and self-reported use of snus were also collected.

The experiment was exempt from evaluation by the Regional Committee for Medical Research Ethics, as advised by an Ethics committee member. We followed guidelines from the Data Protection Official for Research [16]. All information was recorded anonymously. A debrief explained which manipulations participants had been given, and which warning label that is applied today. Resources to official guidelines about snus and health risks were made available.

#### Outcome measures

Perceived severity of health damage associated with the text message was assessed with the question: ‘In your opinion, how severe are the health damages referred to on the warning label?’ (7-point scale, 1-‘very small’ to 7-‘very serious’). Perceived likelihood of health damage following 1 and 10 years of snus usage was measured as: ‘Of 100 persons using snus regularly for 1 (10) years – how many do you think are victim to such health damages?’ (7 ordinal categories, 0–5, 6–10, 11–15, 16–20, 21–25, 26–30, > 30). Expectations of addiction were assessed by the following question: ‘In your opinion, how many weeks does it take to become addicted to snus?’ (7 ordinal categories, 0–5, 6–10, 11–15, 16–20, 21–25, 26–30, > 30). Demographic variables were gender, age, level of education, and snus habits (never, quit or discontinued or former, tried but no regular use, sometimes, regular use).

#### Statistical analyses

For H1 and H3, *severity* and *likelihood of health damage* were averaged to *Risk1* (short term) and *Risk10* (long term) risk perception [9]. The hypotheses were tested by planned comparisons [17] given the specific predictions. For H2, as the severity estimates did not satisfy the normality requirement, the predicted differences between the three outcome measures *severity*, *likelihood at 1 year*, and *10 years* were assessed using a Mann-Whitney U test*.* First, we checked whether gender, age, snus use, and addiction beliefs affected our outcome measures through a repeated measures ANOVA with label as between-group factor, and Risk1 and 10 as within-group factor. IBM SPSS version 23 was used for all analyses. The statistical power of an ANOVA with four groups (*n*’s = 55, 47, 44, and 49) was 85% (given *p* = .05) to detect an effect size of 0.255 (based on group means 4, 4, 4, and 5 yielding a between-groups SD of .43, divided by their common within-group SD of 1.70).

## Results

Descriptive statistics are presented in Table 2. There were no significant differences in demographics (age, gender, education, snus use) between experimental conditions. The ANOVA indicated no gender differences (*M*_*Females*_ = 4.40 vs. *M*_*Males*_ = 3.80), *F*(1, 178) = 2.703, *p* = .102, partial η^2^ = .015, but snus-users rated risks significantly lower than non-users (*M*_*Users*_ = 3.7 vs. *M*_*Non-users*_ = 4.55), *F*(1, 178) = 6.868, *p* = .010, partial η^2^ = .037. Risk increased along with age, in both short- (*r*(188) = .24, *p* = .001) and long-term (*r*(186) = .15, *p* = .042), and with estimates of ease of addiction, (*r*(188) = .16, *p* = .025).

**Table 2:** Study 1: Demographics for participants in each experimental condition. The textual content of warning labels is described in Table 2

Demographic	Cancer (*N* = 55)	Can-can (*N* = 47)	Can-is (*N* = 45)	Will-is (*N* = 49)
Age Mean (SD)	33.40 (9.69)	33.41 (9.51)	34.26 (11.04)	35.22 (12.71)
Gender				
Female	44	40	30	37
Male	9	6	14	10
Missing	2	1	1	2
Education				
High school or less	12	14	11	14
Some college	10	7	15	7
Bachelor’s degree or more	31	24	17	25
Other		1	1	1
Missing	2	1	1	2
Snus habits				
Never	22	21	18	24
Tried or quit	11	12	12	8
Sometimes or regularly	20	13	14	15
Missing	2	1	1	2

*Note*. *SD* Standard Deviation

### H1: EU can vs. will

To test the EU expectation that removing *can* from *damages your health* increases risk perception, we compared the outcome measures *Risk 1* and *10* for the two combined *can* labels (2 and 3 in Table 1) vs. *will* (4 in Table 1). For *Risk 1*, a contrast analysis of the mean scores between these labels did not indicate a significant difference (*M*_Can_ = 3.75 vs. *M*_Will_ = 4.00), *t*(192) =. -1.058, *p* = .291. The corresponding contrast for *Risk10* scores demonstrated higher scores for *will* compared to *can* (*M*_Can_ = 4.37 vs. *M*_Will_ = 4.94), *t*(190) = − 2.135, *p* = .034. These results thus partly supported the EU hypothesis, as removing *can* was associated with a higher risk perception estimates in the long-term (10 years) but not in the short-term (1 year).

### H2: Complex can vs. will

This hypothesis states that *can* labels evoke expectations of higher severity and lower likelihood, whereas a reversed pattern is expected for *will*. To test this, we conducted a non-parametric Mann-Whitney U test for non-normally distributed data, with combined *can* vs. *will* labels as conditions (see above). Neither *severity* (*U* = 2635, *z* = 1.678, *p* = .093), *likelihood 1* (*U* = 2424, *z* = .752, *p* = .452) or *10 years* (*U* = 2606, *z* = 1.899, *p* = .058) were significantly different over the conditions.The mean and median ranks are shown in Table 3. Hence, these results did not support H2.

**Table 3:** Study 1: Risk perception between *can* and *will* labels

	Severity	Likelihood 1 year	Likelihood 10 years
Can			
Mean (SD)	4.37 (1.57)	3.14 (2.13)	4.37 (1.99)
Median (IQR)	4 (2)	2 (4)	4 (3)
Will			
Mean (SD)	4.82 (1.62)	3.24 (1.89)	5.06 (1.67)
Median (IQR)	5 (2)	3 (3)	5 (3)

*Note. SD* Standard Deviation. *IQR* Interquartile Range

Measured on 7-point scales ascending from (1) ‘Very small’ to (7) ‘Very high’ for *severity*, and 0–5, 6–10, 11–15, 16–20, 21–25, 26–30, > 30 victims to health damage for *likelihood*

#### H3: Cancer vs. general health

To test the expectation that an explicit cancer warning is more alarming than the general health versions, we compared the outcome measures *Risk 1* and *10* for the *causes cancer* label (1 in Table 1) vs. *general health* (all other labels). For *Risk 1* and *Risk 10*, contrast analyses of the mean scores between these conditions did not indicate significant differences, *Risk 1* (*M*_Cancer_ = 4.03 vs. *M*_Health_ 3.84), *t*(192) = −.770, *p* = .442, and *Risk 10* (*M*_Cancer_ = 4.8 vs. *M*_Health_ = 4.56), *t*(190) = − 1.049, *p* = .295, respectively. Hence, these results do not support H3.

In sum, the present data did not support the idea that *can* vs. *will* labels affect likelihood or severity perceptions differentially [15], nor that adding *causes cancer* has a stronger effect than general health warnings. However, the results render some support to the EU idea that removing *can* increases risk perception, but only for long-term estimates.

### Study 2

Study 2 added a pre-measure of the outcome variables, increasing the possibility of identifying changes in risk perception levels caused by warning labels. In Study 2 we focused on H1 (removing *can* from *damages your health* strengthens the risk message), and H3 (snus *will* severely damage health and *cause cancer* generates stronger risk expectation than either of the other labels). Also, we added a control group that read the label “Snus” without any warning to examine the effect of repeated risk assessments per se. Eight experts from the tobacco group in the Norwegian Directorate of Health also answered the pre-questionnaire without the warning manipulation, serving as an expert panel for comparison with laymen’s risk perception.

In contrast to Study 1, Study 2 asked participants to rate a number of specific short and long time health hazards following the general risk measures. These estimates can function both as testing knowledge of hazards from snus use, but also as a primer actively reminding participants of possible health hazards. A likely effect of this procedure is that participants demonstrate an overall increased risk perception at post-test. Importantly, if warning labels serve their purpose, their effect should be enhanced by this procedure.

Recruitment procedure, data collection, exclusion criteria, ethical considerations and debriefing were identical to Study 1. No compensation for participation was offered. Participants were recruited through the official Facebook profile of the Department of Psychology, UiT The Arctic University of Norway (*n* = 78), slutta.no, a site for people intending to quit using smoke or snus (*n* = 85), snowballing on the Facebook profile (*n* = 220), and through an internet learning platform for two local high schools (*n* = 123) in February 2016. Eight tobacco experts at the Norwegian Directorate for Health also answered the pre-questionnaire.

Data collection started with a baseline measure of risk perception from snus, both for general health and cancer risk. Next, participants were randomized to read one of five warning labels (four being the same as in Study 1, plus a control group). Following presentation of the labels, participants responded to the same questions as in the baseline.

### Participants

A total of 515 respondents started the survey, 423^2^ completed it_2_. Table 4 presents descriptive statistics for the participants who completed the survey_3_. One respondent reported max values on all measures and was excluded from the analyses.

**Table 4:** Study 2: Demographics for participants in each experimental condition

Demographic	Control (*N* = 104)	Cancer (*N* = 64)	Can-can (*N* = 73)	Can-is (*N* = 100)	Will-is
					(*N* = 82)
Age Mean (SD)	33.65 (12.41)	30.42 (12.70)	31.28 (12.98)	34.15 (14.13)	34.05 (12.42)
Gender					
Female	48	35	34	47	35
Male	18	14	20	27	24
Missing	66	15	19	26	23
Education					
High school or less	14	19	16	23	19
Some college	8	10	7	9	9
Bachelor’s degree or higher	41	16	25	37	31
Other	3	3	4	5	1
Missing	38	16	21	26	22
Snus habits					
Never	25	18	25	38	26
Tried or quit	16	12	11	9	15
Sometimes or regularly	25	18	18	27	18
Missing	38	16	19	26	23

*Note*. *SD* Standard Deviation

### Outcome measures

*General health* was operationalized as ‘In your opinion, how harmful is snus to general health?’, and *cancer* as ‘In your opinion, to what extent does snus cause cancer?’ Both were answered on a 9-point scale ranging from ‘not at all’ to ‘extremely’, and were averaged into a common variable named *Risk perception* corresponding to Popova and Ling [9]*. Specific health hazards 1 year (short-term)* and *30 years (long-term)* listed twelve health hazards from snus use (oral cavity, gaining weight, obesity, diabetes, pregnancy complications, increased heart rate and blood pressure, increased risk of dying from stroke and cardiac arrest, and for developing oral, oesophagus or pancreas cancer). All hazards were extracted from a national report on actual hazards from snus [1]. Estimates were given on a rating scale to the statement “I believe short-term/long-term regular snus use may lead to (12 hazards)” (1: ‘not at all’; 5: ‘extremely’). Finally, participants were presented with all four warning labels and asked to rate which one was the most alarming. *Demographic questions* were identical to Study 1. Snus use were coded into 1) non-users: never tried, quit, tried but no regular use, and 2) users: sometimes, regular but trying to quit, and regular users.

### Design and statistical analyses

Initial analyses were performed to assess the effect on *risk perception* of gender, age, snus use and addiction beliefs. Next, ANOVAs^3^ were used to test planned comparisons [17].When testing H1 and H3, the between-subjects factor was the five different warning labels with *time* as the within-subject (pre-post) factor. The statistical power of a repeated ANOVA with five groups (*n*’s = 73, 100, 82, 64 and 104) was 17% (given *p* = .05 and pre-post *r* = .71) to detect an effect size of 0.068 (given pre-test means 5.68, 5.90, 5.96, 5.89, 5.89 and post-test means 5.72, 6.22, 5.97, 6.03, 6.03 yielding a between group SD = 0.13 divided by their common within-group SD of 1.84).

## Results

The overall ANOVA indicated that females regarded snus use as more risky than males (*M*_*Females*_ = 6.5 vs. *M*_*Males*_ = 4.9), *F*(1, 277) = 33.426, *p* = .000, partial η^2^ = .108, and snus-users regarded snus as less risky compared to non-users (*M*_*Users*_ = 4.5 vs. *M*_*Non-users*_ = 6.8), *F*(1, 277) = 84.030, *p* = .000, partial η^2^ = .233. Also, risk perception tended to increase with increasing age, *r*(299) = .17, *p* = .004 and with estimates of ease of addiction, *r*(422) = .29, *p* = .000. As none of these factors interacted significantly with the outcome measures or with label manipulations, they were not included in the analyses reported below.

### H1: EU can vs. will

The ANOVA demonstrated a significant main effect of time on *Risk perception*, increasing from pre to post, (*M*_*Pre*_ = 5.87 vs. *M*_*Post*_ = 7.13), *F*(1, 418) = 391.46, *p* = .000, partial η^2^ = .484. Levels of risk perception are presented in Table 5. H1, that the *will* label affects risk perception more than the combined *can* labels, was not supported by a contrast analysis of post measures, (*M*_*Can*_ = 6.00 vs. *M*_*Will*_ = 6.03), *F*(1, 418) = .751, *p* = .387.

**Table 5:** Study 2: Mean (SD) for general risk perception before and after seeing warning labels. Measured on 9-point scales ascending from (1) ‘Not at all’ to (9) ‘Extremely’

	Pre	Post
Expert panel	3.38 (1.03)	–
Control	5.68 (1.93)	5.72 (2.05)
Causes cancer	5.90 (2.21)	6.22 (2.24)
Can-can	5.96 (2.11)	5.97 (2.21)
Can-is	5.89 (1.91)	6.03 (1.99)
Will-is	5.89 (1.96)	6.03 (2.15)

*Note*. *SD* Standard Deviation

### H3: Cancer vs. general health

Similarly, H3 (*causes cancer* generates higher risk estimates compared to the other labels combined), was not supported, (*M*_*Cancer*_ = 6.22 vs. *M*_*Others*_ = 6.01), *F*(1, 418) = 1.101, *p* = .315. An overall comparison between all warning labels vs. no warning (control group) indicated no difference, (*M*_*Others*_ = 6.06 vs. *M*_*Control*_ = 5.72), *F*(1, 418) = 0.004, *p* = .947.

When presented all warning labels simultaneously, a majority chose the *causes cancer* warning as most alarming (73%), followed by the *will* warning (17%). Other warnings were < 2%.

### Laymen vs. experts

At baseline, experts perceived the general risk as lower than participants, (*M*_*Experts*_ = 3.38 vs. *M*_*Laymen*_ = 5.86), *F*(1, 428) = 12.178, *p* = .001, partial η^2^ = .028.

### Specific health hazards

The baseline sum score of specific health hazards were significantly lower for short-time vs. long-time ratings of snus use, (*M*_*Short*_ = 33.12 vs. *M*_*Long*_ = 41.10), *F*(1, 419) = 476.80, *p* = .000. The correlation between *Risk perception* and the summed hazards ranged between *r =* .57–.62, indicating that 62–68% of the variance in risk estimates are determined by other factors than perceived health hazards from snus. The tendency was more pronounced among snus-users than non-users, *r*_*Users*_ = .76–.81 vs. *r*_*Non-users*_ = .58–.63.

## Discussion

Two experiments examined risk perception from textual snus warning labels among Norwegian respondents. In Study 1, the new EU-warning (*damages your health*) tended to induce higher long-term (10 years) risk perceptions compared to the former warning moderated by *can*, but these labels did not differ in short-time (1 year) risk estimates. A hypothesis that *can* warnings are associated with more extreme risk perception whereas *will* warnings trigger less serious and more common damages was not supported, neither was the assumption that *causes cancer* generates higher risk perception compared to general health warnings. In Study 2, different text labels did not demonstrate any effect on outcome measures, as risk perception increased similarly over all conditions. In the simultaneous rating of all labels, *will* was perceived as more alarming than *can*. In sum, apart from the fact that these results render some support to the EU’s expectation that removing *can* enhances long-term risk estimates, the effects of textual warnings seem to be negligible.

If the strengthened EU label affects long-term risk perception only, its effect on prevention of snus use among young people may be questioned. The fact that the studies reported here do not demonstrate effects of warning labels in short-time risk estimates, indicates that textual warnings do not affect the main target population well. Focusing on short-time negative consequences rather than serious, long-time consequences might be expected to work more effectively in prevention. From this perspective it is of interest that reading a list of specific and concrete health hazards associated with snus use (Study 2) increased risk estimates significantly. This result indicates that a focus on specific hazards may activate increased awareness about those hazards, which in turn increases risk perception.

The fact that increased risk perception occurred without any differential effects of textual labels in Study 2 may indicate that the repeated hazard estimation questions masked the textual warning manipulations. However, we believe that repeated hazard questions effectively worked as a priming procedure, activating possible negative consequences of snus use and thereby enhancing potential effects of textual warnings. As differential effects of textual warning messages did not appear, we interpret this as even stronger evidence that textual warnings do not affect risk perception. This conclusion agrees with the findings by Popova and Ling [9], who found that snus warning labels, even graphic warnings, did not increase risk perception in non-smokers. As they found positive effects of labels for moist snuff and e-cigarettes, it may be asked if snus warning messages at all affect risk perception.

The tendency for users to rate harmfulness of risks as lower than non-users agrees with the findings of Øverland et al. [18]. They also reported that 41% of Norwegian adolescents rated the harmfulness of snus as equal to or higher compared to cigarettes. This agrees with the fact that our expert panel rated most health risks as lower than the laymen, especially compared to non-users. One explanation for this difference may be that experts are viewing risk in a public health perspective, whereas laymen operate with a personal reference perspective.

### Limitations

The results of Study 2 must be interpreted with some caution, as the statistical power was very low. Given the very small effect size, we cannot expect that increasing the sample size would make much difference as the effect of this intervention would still be minor.

Participants were recruited from social media through e.g. interest groups such as quit intenders, and from high school students. This implies that our sample may be different from the general population, and results should be interpreted with this limitation in mind. Also, the same argument goes for the limited 8-member expert panel, as they may not be representative of all tobacco experts.

A study including risks from other, comparison topics (e.g. smoking, driving, eating chocolate) would have served to place risk ratings of snus usage in perspective. Still, participants in our studies apparently agreed on the risks from snus when judged in isolation. Further, the study could have measured behavioural outcome as well, such as intentions to use or quit snus. Our study only measured risk perception directly after having read a warning label, and did not examine how the different labels might have affected risk perception over time.

## Conclusions

Study 1 found that removing the modal verb *can* from *damages your health* in snus warning labels may affect long-term risk perception, but no heightened risk perception from *causes cancer*. Study 2 did not reveal any differences between labels, but risk perception increased in all conditions, probably due to answering the specific hazard ratings.

## Footnotes

1 Photo of snus product: Rebecca Ravneberg, Norwegian Directorate for Health. The textual warning message on the product is altered across experimental conditions.

2 The sample size for post general risk outcome measures were 423. However, the sample size decreased to 252 for the post specific hazards measures. As descriptive measures were collected at the end of the questionnaire, the sample characteristics only describes those who finished the survey.

3 The randomization worked well as the demographics and pre-test outcome variables *F*(1, 515) = .730, *p* = .572, partial η^2^ = .006 showed no significant differences between the groups. Dropouts did not differ in general risk perception pre-manipulation, compared to those who completed the survey, as shown in a one-way ANOVA with completion/non-completion as predictor *F*(1, 515) = 1.901, *p* = .169, partial η^2^ = .004.

## References

[1] Norwegian Institute of Public Health. Health risks of Scandinavian snus consumption (English summary). 2014. https://www.fhi.no/en/publ/2014/helserisiko-ved-bruk-av-snus/. Accessed 7 Apr 2018.

[2] Levy DT, Mumford EA, Cummings KM, Gilpin EA, Giovino G, Hyland A, Sweanor D, Warner KE (2004). The relative risks of a low-nitrosamine smokeless tobacco product compared with smoking cigarettes: estimates of a panel of experts. Cancer Epidemiol Biomarkers Prev.

[3] Hammond D. Health warning messages on tobacco products: a review. Tob Control. 2011; 10.1136/tc.2010.037630. 10.1136/tc.2010.037630 21606180

[4] Brubaker RG, Mitby SK. Health-risk warning labels on smokeless tobacco products: are they effective? Addict Behav. 1990; 10.1016/0306-4603(90)90014-O. 10.1016/0306-4603(90)90014-o 2343784

[5] MacKinnon DP, Fenaughty AM. Substance use and memory for health warning labels. Health Psychol. 1993; 10.1037/0278-6133.12.2.147. 10.1037//0278-6133.12.2.147 8500442

[6] Agaku IT, Singh T, Rolle IV, Ayo-Yusuf OA. Exposure and response to current text-only smokeless tobacco health warnings among smokeless tobacco users aged ≥18 years, United States, 2012-2013. Prev Med. 2016; 10.1016/j.ypmed.2016.02.014. 10.1016/j.ypmed.2016.02.014 26892913

[7] Fong GT, Hammond D, Hitchman SC. The impact of pictures on the effectiveness of tobacco warnings. B World Health Organ. 2009; 10.2471/BLT.09.069575. 10.2471/BLT.09.069575 PMC2733253 19705020

[8] Adkison SE, Bansal-Travers M, Smith DM, O’Connor RJ, Hyland AJ. Impact of smokeless tobacco packaging on perceptions and beliefs among youth, young adults, and adults in the U.S: findings from an internet-based cross-sectional survey. Harm Reduct J. 2014; 10.1186/1477-7517-11-2. 10.1186/1477-7517-11-2 PMC3942180 24433301

[9] Popova L, Ling PM. Nonsmokers’ responses to new warning labels on smokeless tobacco and electronic cigarettes: an experimental study. BMC Public Health. 2014; 10.1186/1471-2458-14-997. 10.1186/1471-2458-14-997 PMC4190284 25253295

[10] EUR-LEX.europa.eu. Council directive 92/41/EEC of 15 may 1992 amending directive 89/622/EEC on the approximation of the laws, Regulations and administrative provisions of the member states concerning the labelling of tobacco products. 1992. http://eur-lex.europa.eu/legal-content/en/ALL/?uri=CELEX%3A31992L0041. Accessed 7 Apr 2018.

[11] EUR-LEX.europa.eu. Directive 2001/37/EC Manufacture, Presentation and sale of tobacco products. 2001. https://eur-lex.europa.eu/legal-content/EN/TXT/?uri=LEGISSUM%3Ac11567. Accessed 7 Apr 2018.

[12] European Commission. Revision of the Tobacco Products Directive. https://ec.europa.eu/health/tobacco/products/revision/. (n.d). Accessed 31 Mar 2018.

[13] EUR-LEX.europa.eu. Directive 2014/40/EU of the European Parliament and of the council of 3 April 2014 on the approximation of the laws, regulations and administrative provisions of the member states concerning the manufacture, Presentation and sale of tobacco and related products and repealing directive 2001/37/EC text with EEA relevance. 2014. https://eur-lex.europa.eu/legal-content/EN/TXT/?uri=OJ:JOL_2014_127_R_0001. Accessed 7 Apr 2018.

[14] Health Canada. Final report. Health Warning Messages on Smokeless Tobacco, Cigars and pipe products a qualitative study with consumers, in Tobacco Control Programme. 2003. http://www.tobaccolabels.ca/wp/wp-content/uploads/2013/12/Canada-2003-Health-Warning-Messages-on-Smokeless-Tobacco-Cigars-and-Pipe-Products-A-Qualitative-Study-with-Consumers-Government-Report.pdf. Accessed 7 Apr 2018.

[15] Teigen KH, Filkuková P. Can>Will: Predictions of What Can Happen are Extreme, but Believed to be Probable. J Behav Decis Making. 2013; 10.1002/bdm.761.

[16] Data Protection Official for Research. 2016. http://www.nsd.uib.no/personvern/ en/index.html. Accessed 2 May 2016.

[17] Keppel G, Wickens TD (2004). Design and analysis. A researchers handbook.

[18] Øverland SJ, Hetland J, Aarø LE. Relative harm of snus and cigarettes: what do Norwegian adolescents say? Tob Control. 2008; 10.1136/tc.2008.026997. 10.1136/tc.2008.026997 18849315

